# Evolution of an extensively antibiotic resistant sublineage of lineage 1 of GC1 *Acinetobacter baumannii*

**DOI:** 10.1038/s44259-025-00103-5

**Published:** 2025-05-09

**Authors:** Christopher J. Harmer, Ting L. Luo, Francois Lebreton, Patrick T. McGann, Ruth M. Hall

**Affiliations:** 1 School of Life and Environmental Sciences, The University of Sydney, Sydney, NSW 2006 Australia; 2 Multidrug-Resistant Organism Repository and Surveillance Network (MRSN), Bacterial Diseases Branch, CIDR, Walter Reed Army Institute of Research, Silver Spring, MD USA

**Keywords:** Antimicrobial resistance, Pathogens

## Abstract

The multiply antibiotic-resistant lineage 1 of *Acinetobacter baumannii* global clone 1 (GC1) emerged in the 1970s, and subsequently more extensively resistant sublineages have emerged. Here, we examined the evolution of the extensively resistant MRSN56 sublineage and showed it is characterised by insertions carrying resistance genes at specific chromosomal positions. An evolved form of the sublineage carries KL17 replacing KL1 at the capsule locus and includes an additional integrative element Aci-IE1 carrying further resistance genes including *bla*_NDM_. Further members of the modified sublineage (isolated 2014–2021) identified among publicly available genomes were from several countries and appear to have replaced the original form (2007–2010). Some KL17 type isolates had acquired even more resistance genes including *bla*_PER_. The *bla*_NDM_ and *bla*_PER_ genes contribute to reduced susceptibility to cefiderocol and/or sulbactam/durlobactam. The phylogeny indicated that separation of the sublineage into KL1 and KL17 groups coincided with the KL switch and Aci-IE1 was acquired later.

## Introduction

Outbreaks of multiply and extensively antibiotic resistant *Acinetobacter baumannii* are reported worldwide, particularly in nosocomial infections and during periods of societal upheaval such as natural disasters and war^1^. Understanding how this organism establishes itself within patient populations is key to interrupting transmission. The two largest groups of *A. baumannii* isolates belong to two globally disseminated clones known as global clones GC1 and GC2, corresponding to clonal complexes CC1 and CC2 of the Institut Pasteur MLST scheme. In a recent study, ST2 (the main ST for GC2/CC2) represented 63.6% of sequenced genomes and ST1 genomes at 3.6% were the second largest group^2^. However, the contribution of GC1 *A. baumannii* may be greater as ST1 represents only one of several main STs found in GC1/CC1 isolates. The original detailed bioinformatic analysis of available sequences revealed two main lineages of GC1 isolates^3^ although more have been identified since^4–6^. Lineage 1, which is strongly associated with ST1, was estimated to have arisen in the mid to late 1970s coinciding with the first report of an *A. baumannii* resistant to almost all antibiotics available at that time^3^.

The founding event for lineage 1 was the acquisition of a complex transposon designated AbaR0 that carries seven antibiotic resistance genes^3^. AbaR0 jumped into the *comM* gene in the chromosome and AbaR0 or a variant form derived from it is found in all GC1 lineage 1 isolates^7^. Lineage 1 has been highly successful and has spread around the world, being found on all inhabited continents^4^. It has also evolved repeatedly by acquiring resistance to new antibiotics as they were introduced and also by replacing the KL (K locus) and or OCL (outer core locus) regions of the chromosome^3,4,6^. The KL and OCL regions encode the pathways for synthesis of surface polysaccharides, the capsular polysaccharide and the lipooligosaccharide, respectively^8^. Various sublineages of lineage 1 are distinguished by specific events leading to alterations in the structure of surface polysaccharides and/or to the development of resistance to antibiotics introduced later^4,6^. Hence, extensive- or pan-resistance to currently therapeutically relevant antibiotics has developed multiple times.

Recently, an outbreak of an extensively antibiotic-resistant *A. baumannii* strain in three military treatment facilities linked to the conflicts in Iraq and Afghanistan was shown to involve a specific sublineage of GC1 lineage 1^9^. This sublineage consisted of fifty-nine isolates recovered from 30 patients over a 4-year period (2007–2010) that were identified among a large collection of isolates using core genome multilocus sequence typing (cgMLST). These isolates, which were all ST1:KL1:OCL1 or a minor ST1 variant ST1090:KL1:OCL1, differed by fewer than 10 core genome alleles and only 0 to 18 single nucleotide polymorphisms (SNPs). They all carried the same complement of resistance genes, with the exception of the amikacin resistance gene *aphA6* which was present in some isolates but missing in others^9^.

**Fig. 1:**
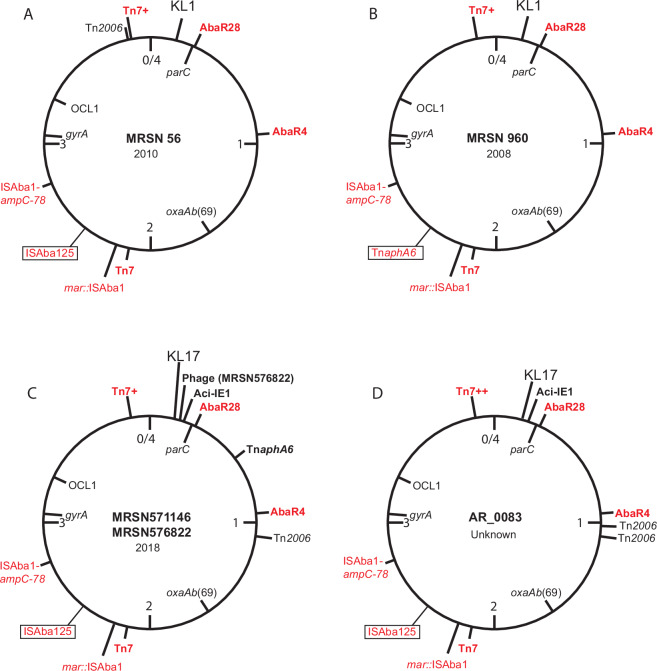
Circular maps of the chromosomes. **A** MRSN56, **B** MRSN960, **C** MRSN571146 and MRSN576822, and **D** AR_0083. Locations of important features e.g. *oxaAb, mar* and *ampC* genes, KL and OCL, and the location of insertions (e.g. AbaR, Tn*7*, Tn*7*+, Tn*7*++, Tn*2006*, Tn*aphA6*, Aci-IE1, and phage) are indicated. Features in red are shared with MRSN56. Drawn to scale from GenBank accession numbers CP080452, JAJEME000000000, CP130627, CP130628 and CP027528 for MRSN56, MRSN960, MRSN571146, MRSN576822 and AR_0083, respectively.

The complete genome of a single isolate, MRSN56 (GenBank accession number CP080452)^10,11^, was used to determine the configuration and location of the resistance genes (Fig. 1A). MRSN56 carried *aphA1, aacC1, aadA1* and *sul1* (conferring resistance to kanamycin/neomycin, gentamicin, streptomycin/spectinomycin, and sulfonamides, respectively) in an AbaR0-derivative, AbaR28. The *oxa23* gene (conferring resistance to carbapenems) is in AbaR4, a *tni* family transposon that targets *comM*^12^. However, in MRSN56, AbaR28 occupies this site and AbaR4 was found in a unique location (Fig. 1A). MRSN56 also carried two copies of Tn*7* (*dfrA1, sat2, aadA1*). One copy, part of an unusual structure designated Tn*7*+^11^, was located in the preferred location for Tn*7* (downstream of *glmS*) and carried additional *tet*(B) (tetracycline) and *sul2* (sulphamethoxazole) resistance genes. A second copy of Tn*7* was in a specific secondary site. Many isolates also carried the amikacin resistance gene *aphA6* in Tn*aphA6* which was traced to a specific location in the chromosome of an amikacin resistant isolate MRSN960 (location marked in Fig. 1B). The complete sequence of MRSN56 also revealed a novel route to fluroquinolone resistance that appears to involve a mutation in *gyrA* combined with inactivation of the chromosomal *marR* gene in the *mar* operon by ISAba1 (Fig. 1A) leading to constitutive expression of *marA* from the promoter internal to ISAba1^11^. An ISAba1 9 bp upstream of the chromosomal *ampC* gene that confers 3^rd^-generation cephalosporin resistance was also present but the *ampC* allele had been replaced as seen previously in other GC1 sublineages^3^. MRSN56 also carried an additional 17 copies of ISAba1 in the chromosome.

The previous study^9^ included two additional isolates MRSN571146 and MRSN576822 that were cultured from wounds of two Afghan civilians at a US military facility in Afghanistan some years later in 2018. In the cgMLST analysis these isolates were more distantly related to the 59 KL1 isolates in the MRSN56 group differing by 13–21 core genome alleles and 32–47 SNPs. They carried KL17 replacing KL1 at the capsule biosynthesis K locus. They also carried two additional antibiotic resistance genes not found in the rest of the collection, *bla*_NDM-1_ conferring resistance to all β-lactams except the monobactams, and *strA* conferring resistance to streptomycin^9^.

The identification of MRSN571146 and MRSN576822 from 2018 that grouped with the rest of the collection but differed at the K locus and in their antibiotic resistance gene content, suggested these isolates may represent further evolution of this sublineage via acquisition of additional resistance genes and a KL switch via recombination. Here, we have completed the genome of these two isolates and determined the context of their additional antibiotic resistance genes. We also examined the key features and relationship to MRSN571146, MRSN576822 and MRSN56 of the known MRSN56 relatives. In public databases we identified genomes from isolates that were closely related to MRS571146 and MRSN576822, recovered in 11 different countries. The context of each gene in the resistance gene repertoire was analyzed and acquisition and loss events correlated with the phylogeny. The locations of ISAba1 copies not associated with antibiotic resistance were also examined.

## Results

### Complete genomes of MRSN571146 and MRSN576822

To facilitate the examination of resistance islands and other features of the MRSN571146 and MRSN576822 isolates, the complete genomes were determined. The Trycycler hybrid assembly for MRSN571146 produced a circular chromosome and a 124.5 kb closed circle that included a segment of the standard GC1 chromosome that is flanked by directly oriented ISAba1 copies in MRSN56. This segment was incorporated at the standard location to generate a 4,020,125 bp circular chromosome (GenBank accession number CP130627). An 8731 bp Rep_3 plasmid in the R3-T1 group that is identical to pA1-1 (GenBank accession number CP010782) found in an early GC1 lineage 1 isolate^13^ and pMRSN56-4 (GenBank accession number CP080456) was also present. MRSN571146 does not include the three other plasmids found in MRSN56. The hybrid assembly of MRSN576822 (GenBank accession number CP130628) produced a 4,058,653 bp circular chromosome after incorporation of the same 124.5 kb segment but no plasmids. The difference in size between the MRSN571146 and MRSN576822 chromosomes is due to the presence of a 37,244 bp phage genome located between locus tags K9C73_12640 and K9C73_12945 in MRSN571146 (location marked in Fig. 1C) and an additional copy of ISAba1 in MRSN576822. The prophage is unique to MRSN576822 and was not found in other isolates in the sublineage. It was found in only a single other complete *A. baumanni* in GenBank (GenBank accession number CP050403).

**Fig. 2:**
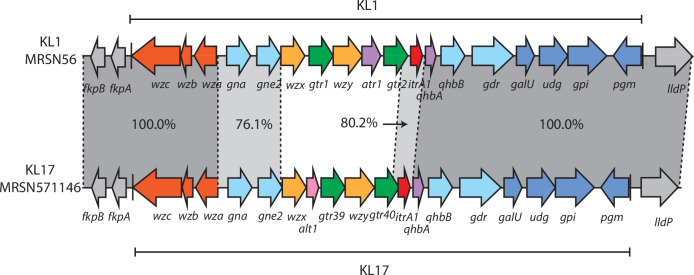
Linear comparison of KL1 from MRSN56 and KL17 from MRSN571146. Dark shaded areas indicate 100% nucleotide identity, lighter shading indicates >75% nucleotide identity. The extent of the KL is indicated by thin vertical lines. Drawn to scale from GenBank accession numbers CP080452 and CP130627 for MRSN56 and MRSN571146, respectively.

As MRSN571146 and MRSN576822 carry KL17 rather than KL1 at the K locus^9^ they are are ST1:KL17:OCL1 type. As they share many features with MRSN56 and the MRSN56 cluster as described below, this suggested that a KL switch via a recombination patch may have occurred. An alignment with the MRSN56 sequence located a short recombination patch of 7 kbp that lies internal to the K locus (Fig. 2). Consistent with this, the sequence of the surrounding chromosome in MRSN571146 and MRSN576822 was identical to that of MRSN56. Replacement of the set of genes at the K locus leads to production of a different capsular polysaccharide on the cell surface.

### Chromosomal genes conferring resistance

The resistance profiles of MRSN571146 and MRSN576822 were reported previously^9^ and the causes of resistance were examined here. As for MRSN56, a copy of ISAba1 9 bp upstream of the *ampC*-78 allele in the appropriate orientation to enhance expression of *ampC*, accounts for resistance to ceftazidime and cefotaxime^14,15^. The *oxa23* and *bla*_NDM_ carbapenem resistance genes would also contribute to resistance to third generation cephalosporins. The *ampC*-78 allele differs from *ampC*-1 found in most GC1 isolates^3^ and appears to have been acquired by homologous recombination as described previously for MRSN56^11^. Another ISAba1 copy in *marR*, which inactivates the MarR repressor and provides a replacement promoter internal to the ISAba1 to express *marA*, is predicted to confer fluroquinolone resistance when combined with an S81L substitution in GyrA^11^.

### Acquired antibiotic resistance genes

Most of the acquired antibiotic resistance genes in the MRSN571146 and MRSN576822 genomes were distributed among the same transposons (genomic resistance islands) in the same chromosomal location as found in MRSN56 (Fig. 1A). Hence, AbaR28 (*aphA1, aacC1, aadA1* and *sul1*)^10,11^, AbaR4 carrying *oxa23* in Tn*2006*, Tn*7* (*dfrA1, sat2* and *aadA1*) and Tn*7+* carrying *dfrA1, sat2, aadA1, tet*(B) and *sul2* were all present (Fig. 1C). A second copy of Tn*2006* in the MRSN571146 and MRSN576822 chromosomes was not in the same location as the second copy of Tn*2006* found in MRSN56 (compare Fig. 1A, C). This is consistent with a scenario in which the initial entry of Tn*2006* into this lineage was in AbaR4, and this was followed by movement of Tn*2006* on different occasions to additional chromosomal locations found in MRSN56 or MRSN571146 and MRSN576822. The *aphA6* gene, conferring resistance to amikacin, is found in a copy of Tn*aphA6*^16^ located between AbaR28 and AbaR4 (Fig. 1C). However, a copy of ISAba125 (boxed) remains at the location of the Tn*aphA6* (boxed in Fig. 1B) in MRSN960.

The additional antibiotic resistance genes, namely *bla*_NDM-1_, *strAB* and a second copy of *sul2*, found in MRSN571146 and MRSN576822 were not present in any of these regions. Notably, *bla*_NDM_ provides a greater spectrum of β-lactam resistance than *oxa23* and can contribute to reduced susceptibility to cefiderocol^17,18^. Hence, the MIC for cefiderocol was determined and found to be 2 μg/ml for MRSN571146 and MRSN576822 compared to ≤0.25 μg/ml for MRSN56 (break points S ≤ 4, I = 8, R ≥ 16). In addition, MRSN571146 and MRSN576822 were resistant to sulbactam/durlobactam (MIC = 64 μg/ml) whereas MRSN56 was susceptible (MIC = 2 μg/ml)

### A novel genomic island Aci-IE1 in MRSN571146 and MRSN576822

**Fig. 3:**
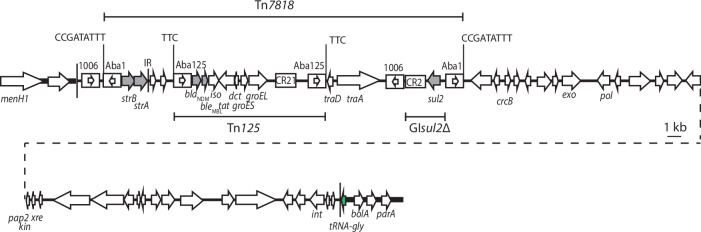
Novel genomic island Aci-IE1. Horizontal arrows show the extent and direction of reading frames with names above and below. The *tRNA-gly* gene targeted by the genomic island is shown in green. Complete and partial copies of insertion sequences and CR2 are shown as boxes with names above. An internal arrow indicates the IS orientation. Target site duplications (TSD) are shown as sequences above vertical lines. The extent of the genomic island is indicated by thin vertical lines. The sequence belonging to the surrounding chromosome is shown as a thick horizontal line.

The locations of *bla*_NDM-1_, *strAB* and the second copy of *sul2* were found by comparing the chromosomes of MRSN571146 and MRSN576822 to that of MRSN56. This identified a 61,445 bp segment (Fig. 3) that included these resistance genes and was located in a tRNA-gly gene between KL17 and *parC* (Aci-IE1 in Fig. 1C). The resistance genes are all contained in a transposon bounded by inversely oriented copies of ISAba1, here named Tn*7818*, which is surrounded by a 9 bp target site duplication (CCGATATTT) (Fig. 3). The *sul2* gene is in a fragment derived from GI*sul2*^19^, and the *bla*_NDM_ carbapenem resistance gene is in Tn*125* (Fig. 3). The *traA* and *traD* transfer genes suggest a plasmid origin but do not match any known transfer genes.

The backbone of this island appears to be an integrative element (IE) as it includes an *int* gene (Pfam PF00589) encoding a site-specific recombinase of the tyrosine recombinase family (Fig. 3). The IE, named Aci-IE1 for *Acinetobacter* integrative element 1, includes 76 nucleotides corresponding to bases 171324-171399 in MRSN56 (GenBank accession number CP080452) thereby re-forming a complete tRNA-gly and creating a 76 bp duplication. Searches of complete *A. baumannii* genomes in GenBank revealed that this IE is found in only one complete GC1 genome, AR_0083 (GenBank accession number CP027828) that is described in more detail below. However, it is found in the same location in four GC2 isolates (VB473, VB958, VB7036 and VB1136; GenBank accession numbers CP050388, CP040040, CP050525 and CO040047, respectively) all recovered in India. In all instances, the complete island including the ISAba1-bounded Tn*7818* transposon is present. The backbone without resistance genes was not seen. The DNA sequence surrounding the Aci-IE1 island in the GC2 genomes is identical to that of the GC2 reference strain A320 (GenBank accession number CP032055)^20^, indicating that this is an independent acquisition of Aci-IE1 and not the result of recombinational exchange between a GC1 and a GC2 strain.

### Isolate AR_0083

Searches of the GenBank non-redundant database using two of the key features found in MRSN56, MRSN571146 and MRNS576822 that potentially define the MRSN56 sublineage, namely the left-hand boundary of the AbaR4 antibiotic resistance island and the presence of Tn7+ in the preferred location downstream of the chromosomal *glmS* gene, also identified the genome of AR_0083 noted above as the only complete GC1 genome carrying the novel Aci-IE1 genomic island. AR_0083 was also found to be an ST1: KL17:OCL1 isolate. It also includes one plasmid, an 8731 bp Rep_3 plasmid (CP027529) that is identical to pA1-1 (CP010782) and is also found in MRSN56 and MRSN571146. The source and year of isolation of this isolate is not recorded but it is available from the CDC ARIsolateBank (www.cdc.gov/ARIsolateBank) and is recorded there as resistant to ampicillin/sulbactam, piperacillin/tazobactam, the carbapenems imipenem, meropenem and doripenem, and the third and fourth generation cephalosporins cefotaxime, ceftazidime, ceftriaxone and cefepime. It is also resistant to fluoroquinolones, ciprofloxacin, levofloxacin and moxiflocaxin, and aminoglycosides amikacin, gentamicin and tobramycin as well as to trimethoprim-sulphamethoxazole and tetracycline. It was susceptible/reduced susceptibility to only tigecycline and colistin. Hence, it resembles MRSN56, MRSN571146 and MRSN576822 but is also resistant to tobramycin.

The AR_0083 chromosome was found to include all of the characteristic insertions found in MRSN56 and in MRSN571146 and MRSN576822 including AbaR28, AbaR4 and Tn*7* as well as the copy of ISAba1 upstream of the *ampC*-78 allele and the copy in *marR* that is predicted to confer fluroquinolone resistance (Fig. 1D). Aci-IE1 was also present (see above). In addition to Tn*2006* in AbaR4, AR_0083 also has two additional copies of Tn*2006* one of which is in a position shared with MRSN571146 and MRSN576822 (Fig. 1C) indicating shared history. Although AR_0083 was amikacin resistant, like MRSN56 it lacked Tn*aphA6* (amikacin resistance) but retained an ISAba125 in that location. However, additional resistance genes were detected, for example, the recorded amikacin and tobramycin resistance are explained by the presence of the *armA* gene that confers resistance to all therapeutically relevant aminoglycosides (see below).

### Tn7++ replaces Tn7+ in AR_0083

**Fig. 4:**
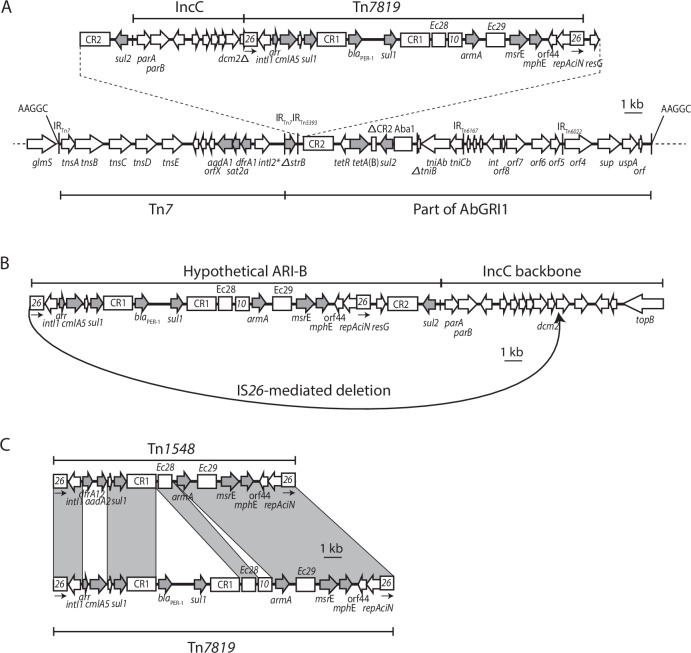
Tn*7*++. **A** Structure of Tn*7*++. **B** Potential route to creation of Tn*7*++ from a hypothetical IncC ARI-B island. **C** Linear comparison of the novel PTn*7819* to Tn*1548*. Horizontal arrows show the extent and direction of reading frames with names above and below. Antibiotic resistance genes are shaded. Complete and partial copies of insertion sequences and CR2 are shown as boxes with names above, with an arrow below copies of IS*26* to indicate the IS orientation. Target site duplications (TSD) are shown as sequences above vertical lines and inverted repeats are denoted by thin vertical lines. Known structures are marked above. Shading indicates shared regions between PTn*7819* and Tn*1548*.

Tn*7* and Tn*7*+ were present in AR_0083 in the locations found in MRSN56 and in MRSN571146 and MRSN576822. However, further examination revealed that an additional 34.6 Kbp segment had been incorporated into Tn*7*+ to yield larger resistance island named Tn*7*++ (Fig. 4A). The additional segment includes a pseudo-compound transposon (PCT) bounded by directly oriented copies of IS*26* that carries the *armA* and *msrE-mphE*, *cmlA5, arr, sul1* (two copies) and *bla*_PER-1_ genes. This PCT, named PTn*7819*, is related to Tn*1548* and Tn*6180*^21,22^. It differs from Tn*1548* by replacement of the *dfrA12* and *aadA2* gene cassettes with *arr* and *cmlA5*, acquisition of a copy of IS*10*, and acquisition of *bla*_PER-1_ and a second copy of *sul1* via homologous recombination within CR1 (Fig. 4C). The *armA* gene adds resistance to all aminoglycosides including tobramycin, the *msrE-mphE* genes add resistance to macrolides, and the *arr* gene adds resistance to rifampicin. In addition, the *bla*_PER-1_ gene has been shown to contribute to resistance to the recently introduced cefiderocol^17,18^, and AR_0083 may be resistant.

However, the additional segment is much larger as it also includes *sul2* and part of the backbone of IncC plasmids that is found adjacent to the ARI-B resistance island which often includes IS*26* copies^23,24^. The route to incorporation of this additional segment into Tn*7*+ to form Tn*7*++ is most simply explained by IS*26*-mediated formation of a translocatable unit (TU) via *in cis* action causing an adjacent deletion (Fig. 4B). The circular TU produced was then incorporated, most likely by homologous recombination between the copy of CR2 in the TU and one in Tn*7*+ as shown in (Fig. 4A). This IS*26*-mediated route to acquisition of resistance genes is described in detail elsewhere^22^.

### Additional MRSN56 sublineage members in the draft genome database

As AR_0083 was the only additional complete genome found with AbaR4 in the location characteristic of the MRSN56 sublineage, the GenBank Whole Genome Shotgun (WGS) database was queried with the sequence of the left and right AbaR4 boundaries to determine if any draft genomes belonged to the MRSN56 sublineage. The 58 KL1 draft genomes reported previously^9^ were all detected using this approach indicating that these features are characteristic of the MRSN56 sublineage. A total of 31 additional genomes were also identified (Table 1), of which 27 were ST1:KL17:OCL1 and 4 were KL1 isolates additional to the MRSN isolates reported previously. The 27 KL17 isolates were recovered between 2014 and 2021, later than the KL1 isolates (2008–2010), indicating that the KL17 type had replaced the KL1 type. The KL17 isolates were recovered in Iraq, Lebanon, Egypt, India, Pakistan, Nepal, USA and South Africa, indicating a more widespread distribution of this sublineage.

**Table 1:** Additional GC1 *A. baumannii* in GenBank with characteristic AbaR4 location

Isolate	Year	Country	KL	OCL	Tn7 + /Tn*7* + +	Tn7	Aci-IE1	ISAba1-*ampC*	ISAba1-*marR*	Accession no.
MRSN571146	2018	Afghanistan	17	1	+	+	+	+	+	CP130627
MRSN576822	2018	Afghanistan	17	1	+	+	+	+	+	CP130628
AR_0083	–	–	17	1	++	+	+	+	+	CP027528
ZQ5^a^	2016	Iraq	17	1	++	+	−	+	+	PHJX00000000
ZQ6^a^	2016	Iraq	17	1	++	+	−	+	+	PHJW00000000
SP3561	2019	India	17	1	+	+	+	+	+	JAAOSU010000000
SP4673	2019	India	17	1	+	+	+	+	+	JAAOSB010000000
2020GN	2020	USA	17	1	+	+	+	−	−	ABEWDL010000000
2020HL466	2020	USA	17	1	+	+	+	+	−	AAYLPN010000000
2020HL469	2020	USA	17	1	+	+	+	+	−	AAYLQK010000000
2021AS	2021	USA	17	1	+	+	+	+	+	ABDOLC030000000
ARLG8183	2019	Lebanon	17	1	+	+	−	−	−	DAKMFX010000000
Aci00879	2017	South Africa	17	1	++	+	+	+	+	CACRXQ010000000
Aci00880	2017	South Africa	17	1	++	+	+	+	+	CACRYG010000000
Aci00881	2017	South Africa	17	1	++	+	+	+	+	CACRXX010000000
Aci00882	2017	South Africa	17	1	+	+	+	+	+	CACRXT010000000
M476 (FG121)	2017	South Africa	17	1	++	+	+	+	+	JAGSHW000000000
DTU2021^b^	2020	Pakistan	17	1	+	+	+	+	+	DAKCBM010000000
IOMTU489^b^	2014	Nepal	17	1	+	+	+	+	−	DADBKQ010000000
IOMTU671^b^	2014	Nepal	17	1	+	+	+	+	−	DADBOE020000000
IOMTU659^b^	2014	Nepal	17	1	+	+	+	−	−	DADBOF020000000
IOMTU666^b^	2014	Nepal	17	1	+	+	+	−	−	DADBOH020000000
A1817^c^	2018	Egypt	17	1	+	+	+	+	−	DAFIDQ010000000
A1818^c^	2018	Egypt	17	1	+	+	+	−	−	DAFIHW010000000
A1826^c^	2018	Egypt	17	1	+	+	+	+	−	DAFIDT010000000
A1827^c^	2018	Egypt	17	1	+	+	+	+	−	DAFIEH010000000
A1828^c^	2018	Egypt	17	1	+	+	+	−	−	DAFIDY010000000
A1830^c^	2018	Egypt	17	1	+	+	+	−	−	DAFIDU010000000
Abau471	2016	Togo	17	1	+	+	+	+	+	NXGU00000000
Abau446	2016	Togo	17	1	+	+	+	+	+	NRID00000000
ABNIH6	2009	USA	1	1	+	+	−	+	+	APAX00000000
ABNIH11	2009	USA	1	1	+	+	−	+	+	APBA00000000
ABNIH19	2009	USA	1	1	+	+	−	+	+	APBH01000000
AB5197	2008	USA	1	1	+	+	−	+	+	LREO00000000

^a^Reported in ref. ^39^.

^b^Reported in ref. ^30^.

^c^Reported in ref. ^31^.

The precise configurations of the resistance islands in the 27 additional KL17 isolates could not be determined as they were split into multiple contigs in the draft assemblies due to multiple insertion sequences (IS) and repeat units. However, the resistance gene content of each isolate was determined using AMRFinderPlus, and the resistance gene configuration and resistance island boundaries in the available contigs were examined. This analysis revealed that in addition to AbaR4 containing *oxa23* in Tn*2006*, they had AbaR28 in *comM* with the characteristic adjacent deletion (Table 1). Some isolates had additional copies of Tn*2006* elsewhere in the chromosome, identified by finding normally contiguous chromosomal regions that were split into two contigs in the draft assemblies with remnants of inversely oriented copies of ISAba1 at the contig ends and matching 9 bp target site duplications (TSD).

All 27 KL17 isolates had two copies of Tn*7*. Either Tn*7*+ or Tn*7*++ was located downstream of *glmS* and Tn*7* was in the secondary site (see Fig. 1 for locations). Only six of the isolates (all from South Africa or Iraq) had the Tn*7*++ arrangement seen in AR_0083. 24 of the 27 additional isolates had the novel Aci-IE1 genomic island containing *strAB, bla*_NDM_ in Tn*125*, and *sul2* (Fig. 3) in the same location as in MRSN571146 and MRSN576822. Tn*aphA6*, identified by recovering a contig consisting of the central portion of the transposon, was present in 25 of the 27 isolates (only absent in 2020HL466 and 2021AS) but the precise chromosomal location could not be confirmed in the drafts as they all had multiple copies of ISAba125. Unexpectedly, *marR* was interrupted by ISAba1 in only 13 of the isolates, and 20 of the 27 had a copy of ISAba1 upstream of *ampC*. Examination of the contigs containing *ampC* or *marR* in the 7 isolates that lacked both IS revealed that the contig was unbroken where the ISAba1 was expected to be and the sequence did not otherwise differ from that surrounding the ISAba1 copies in the MRSN571146 and MRSN576822 isolates.

### The KL17 isolates represent a defined subgroup

**Fig. 5:**
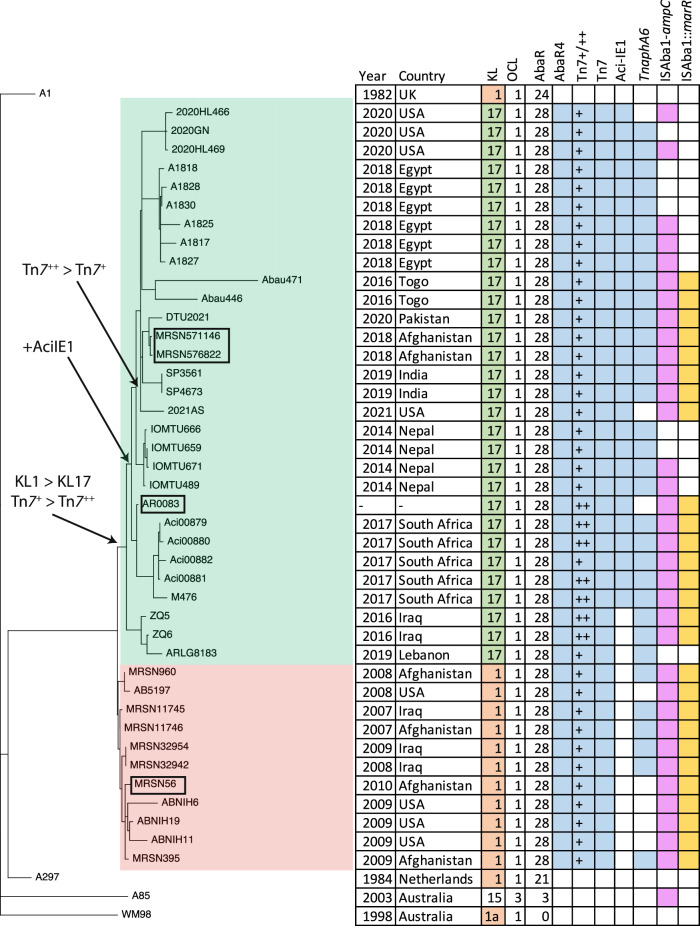
Midpoint-rooted SNP-based phylogeny and key features of the KL1 and KL17 isolates. The early GC1 isolate A1 (GenBank accession number CP010781) was used as the out-group and three additional GC1 lineage 1 genomes, A85 (GenBank accession no. CP021782), WM98 (GenBank accession no. CP116387) and A297 (FBWR00000000), were used to give further topology to the tree. KL1 isolates are shaded red and KL17 isolates are shaded green. Major events including the KL1-KL17 switch, the acquisition of Aci-IE1 and the acquisition/loss of Tn*7*++ are marked. A colored box indicates the presence of that feature.

To examine the evolutionary relatedness of the KL1 and KL17 isolates identified here, a core SNP phylogeny was generated using Snippy (Fig. 5). As we had previously reported a full SNP-based phylogeny of the 59 KL1 MRSN isolates (see Fig. 1 in ref. ^9^), a sub-set that represented one isolate from each year (2007–2010) from both Iraq and Afghanistan was selected. The early GC1 isolate A1 (recovered in 1982, GenBank accession number CP010781) was used as an out-group and three additional GC1 lineage 1 genomes, A85 (GenBank accession no. CP021782), WM98 (GenBank accession no. CP116387) and A297 (FBWR00000000), give further topology to the tree. The KL17 and KL1 isolates formed two distinct but closely related groups (Fig. 5) that were separated by fewer than 50 SNPs in total. The KL switch and the acquisition of the Aci-IE1 genomic island coincided with the major branch points in the tree. Interestingly, Tn*7*++ is represented in two separate branches (the branch containing the Aci and M476 isolates from South Africa, and the branch containing ZQ5, ZQ6 and ARLG8183), indicating that the additional segment may have been acquired early and then lost via recombination between the two copies of CR2. Tn*7*++ is also absent from Aci00882 but present in Aci00879, Aci00880, Aci00881 which were all recovered in 2017 in South Africa, consistent with loss via homologous recombination.

### Locations of ISAba1 copies

**Fig. 6:**
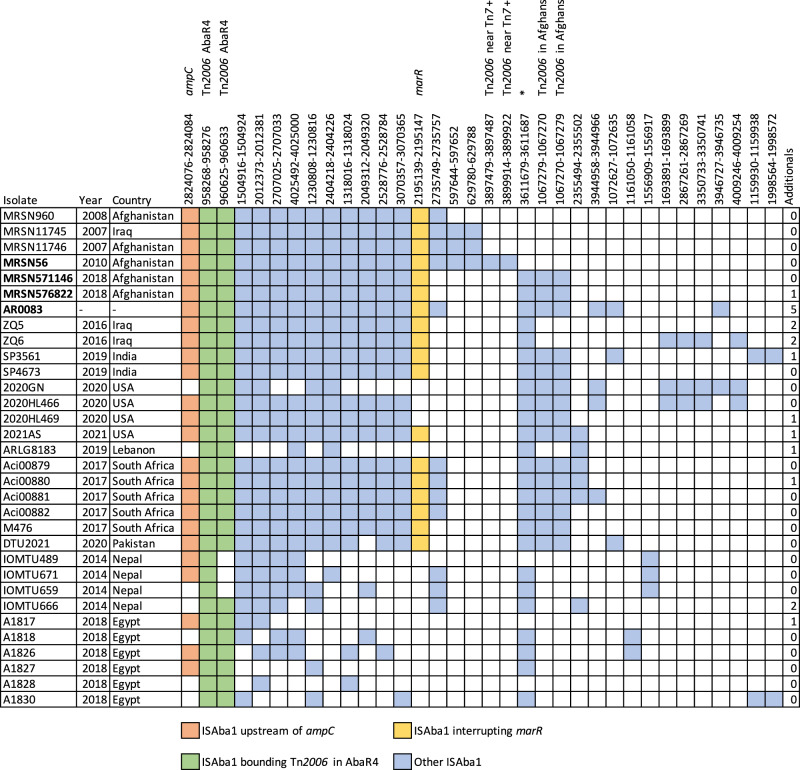
Distribution of ISAba1 in the MRSN56 sublineage. Shading, according to the key below, indicates the presence of a copy of ISAba1 at that location. The position of the 9-bp target site duplication in the MRSN56 genome (GenBank accession number CP080452) is shown above each IS column. The number on the last column indicates the number of unique copies of ISAba1 in that genome.

The specific locations of IS are indicative of shared ancestry involving the movement of the IS to a specific new site and we have previously used the location of ISAba1 copies to distinguish specific GC1 lineages and sublineages^6^. Hence, ISAba1 positions in MRSN571146, MRSN576822 and AR_0083 were located using ISMapper^25^ and compared to locations found previously in MRSN56^11^. Their positions are shown in Fig. 6. The KL17 MRSN571146, MRSN576822, and AR_0083 isolates share 12 of the 15 ISAba1 copies previously identified in MRSN56 (see Fig. 1a in ref. ^11^) that were not found in resistance islands or as part of Tn*2006*, hereafter referred to as solo ISAba1 copies. An additional solo copy of ISAba1 inserted at 361679-88 in MRSN56 was common to MRSN571146, MRSN576822 and AR_0083.

Between 5 and 25 copies of ISAba1 were identified in each of the 25 additional KL17 isolates for which read data was available (Fig. 6). However, only 12 of the 25 had copies of ISAba1 in the 12 shared locations and all of those include the extra ISAba1 found in MRSN571146, MRSN576822, and AR_0083. Most of them also carry Tn*2006* in the location found in MRSN571146, MRSN576822 and AR_0083. Surprisingly, many of the 12 conserved IS locations were not found in the isolates from Egypt, Lebanon, and Nepal plus a single isolate from the USA. This includes the copies upstream of *ampC* and in *marR*, both of which contribute to the resistance profile. Some isolates shared as few as 5 ISAba1 positions with MRSN56, suggesting multiple recombination events may have removed blocks of IS. However, if this is the case a recombination patch has removed the ISAba1 the incoming sequence was identical to the resident one.

## Discussion

Defining steps in the evolution of the MRSN56 sublineage of the GC1 lineage 1 examined here have involved first the replacement of the central region of the K locus leading to production of K17 type CPS rather than K1 type and both the acquisition and the loss of genes that confer resistance to clinically relevant antibiotics in or from regions associated with the chromosome. Plasmids have not played a role. A small Rep_3 plasmid identical to pA1-1, the R3-T1 plasmid which is found in the earliest GC1 isolate for which a sequence is available^13^, was found in all isolates examined except MRSN576822. This plasmid carries no antibiotic resistance genes but our initial investigations indicate that it may be found in most GC1 lineage 1 isolates. Whether this is so can potentially be determined rapidly using the new plasmid typing tool^26^.

Founding steps for the MRSN56 sublineage involved the loss of part of AbaR3 including the *tet*(A) tetracycline resistance gene to form AbaR28^7^ and restoration of tetracycline resistance when the Tn7^+^ carrying the *tet*(B) gene was introduced. The acquisition of AbaR4 carrying the *oxa23* carbapenemase gene and of Tn7 at the secondary site were also founding events and their locations are among the defining features of the MRSN56 sublineage. Many in both the KL1 and the KL17 groups also carried *aphA6* in Tn*aphA6* which supplies resistance to amikacin. However, the *aphA6* gene was not stably maintained in the original location although a copy of ISAba125 was (boxed in Fig. 1) making it another potential defining feature of the sublineage.

All isolates in the MRSN56 sublineage carry the *oxa23* gene making them carbapenem resistant and the addition of *bla*_NDM_ and *bla*_PER_ further reduces susceptibility to more recent options. All isolates in the sublineage included the *aacC1* gentamicin resistance gene which is found in AbaR28 leaving only amikacin and tobramycin as possible suitable aminoglycoside treatment options. However, amplification of the *aphA1* gene (found in AbaR28) is known to confer resistance to tobramycin^10,27^ and the *aphA6* gene, when present, confers resistance to amikacin. The *armA* gene conferring resistance to gentamicin, amikacin and tobramycin appears to have been introduced early in the evolution of the KL17 form of the sublineage but like *aphA6* it was not stably maintained and is found in only a few isolates. In both cases of instability, loss was likely due to homologous recombination between identical directly oriented sequences in the surrounds. Acquisition of the novel Aci-IE1 integrative element carrying the *bla*_NDM_ gene identified here was a later event in the evolution of the KL17 branch.

The replacement of KL1 by KL17 appears to coincide with an increased ability to spread. Whereas the KL1 branch of the MRSN56 sublineage was strongly associated with conflicts in Iraq and Afghanistan^9^, the KL17 branch has clearly spread more widely. The earliest isolates in the KL17 branch were recovered in Nepal in 2014, some 4 years after the last of the KL1 branch isolates were recovered. Hence, the events that distinguish the KL17 branch may have occurred in that period. The presence the KL17 branch in South Africa in addition to countries neighboring Iraq and Afghanistan indicates a wider dissemination of this GC1 sublineage. A further 4 isolates with features of this evolved version of the sublineage were found in among the *A*. *baumannii* carrying *bla*_NDM_ recovered in Mayotte and Reunion Island in 2019^28^. The genome data for these isolates was trimmed precluding their inclusion in Table 1. In the future, as further GC1 genomes become available, it should be possible to determine if the KL17 branch has spread even further. In addition, whether the change in KL, which would alter the structure of the capsular polysaccharide, is directly involved in the displacement of the KL1 branch remains to be examined.

The method we used to find additional examples of genomes belonging to the KL1 and KL17 branches of this sublineage relies on knowledge of where acquired regions that include antibiotic resistance genes are located. This approach is powerful as it can be applied to draft genomes enhancing what can be deduced about relationships between isolates in a way that simply listing the repertoire of resistance genes cannot. However, it relies on the availability of high-quality draft genome data and can be difficult to apply if contig ends have been trimmed to remove repeated segments as is often the case since the introduction of cgMLST. The application of IS mapping to track relationships within this sublineage gave some unexpected results. Positions of copies of ISAba1 are expected to be stable and this has been used for decades to identify related isolates within a single species. IS position stability was also largely the case in previous studies that used this approach^6,29^. Here, the shared IS locations were conserved (and added to) in many of the genomes examined. The MRSN571146 and MRSN576822 isolates share 12 of the 15 solo copies of ISAba1 previously found in MRSN56 (see Fig. 1a^11^), and AR_0083 includes 13 of them. The copy at 361679-88 is present in all but three of the KL17 group. However, in some isolates, mainly derived from two studies^30,31^, multiple ISAba1 copies were not detected and may have been lost. The missing IS included ones that confer resistance to cephalosporins and to fluoroquinolones. At this stage it is not clear whether this reflects an ability to remove IS copies using a currently unknown mechanism or if it is a product of the way that the read data (which is used to map IS positions) has been manipulated. The latter possibility is supported by the finding that, whereas most isolates in the data set examined here retained the ISAba125 found at the location of Tn*aphA6* in MRSN960 some of the isolates that lacked ISAba1 copies also lacked this ISAba125. We have previously encountered missing IS in a single group of sequences from the same study^29^. Further work is needed to resolve this issue granted the power of information on transposon and IS locations in tracing the ancestry of specific lineages and sublineages of important clonal complexes and in tracking ongoing spread.

## Materials and methods

### Genome sequencing, assembly and annotation

Draft assemblies of the MRSN571146 and MRSN576822 isolates were obtained previously from short-read Illumina MiSeq data^9^. Further, genomic DNA was extracted from MRSN571146 and MRSN576822 using the DNeasy UltraClean Microbial Kit (Qiagen). Briefly, a 10 µl loopful of bacteria was removed from an overnight Blood agar culture and DNA extracted exactly as described by the manufacturer. DNA quantity and quality was analyzed using a Qubit 4 Fluorometer (Invitrogen) and NanoDrop spectrophotometer, respectively. Native-barcoded genomic DNA libraries were created using the SQK native barcoding kit v14 and sequenced using a Minion R10.4.1 flow cell per the manufacturer’s instructions. The sequencing of MRSN571146 generated 286,709 reads with an average read length of 1621 bp (116x coverage). The sequencing of MRSN576822 generated 20,858 reads with an average read length of 12,400 bp (62x coverage). Read quality was validated using FastQC (https://qubeshub.org/resources/fastqc).

The MinION reads were first filtered using Filtlong version 0.2.1 (https://github.com/rrwick/Filtlong) to remove reads shorter than 1000 bp and this raised the average read length for MRSN571146 to 25.5 kb and MRSN576822 to 23.9 kb. The output was than reduced to 500 Mbp of long reads. The MRSN571146 and MRSN576822 long reads were combined with the previously obtained short reads^9^ using Trycycler version 0.5.5^32^ with default settings to produce a hybrid assembly. Assemblies were checked for completeness and contamination using CheckM^33^. Protein coding, rRNA and tRNA genes were annotated using Prokka (version 1.12)^34^. Antibiotic resistance genes were identified using AMRFinderPlus^35^, and resistance regions were identified and annotated manually using an in-house database of standard sequences. Plasmids were identified and typed using the Acinetobacter Plasmid Typing scheme version 2.0^26^.

### Identification of genomes containing AbaR4 in the lineage-specific location

Genomes containing AbaR4 in the characteristic location defined in MRSN56^11^ were identified by performing a BLASTn search (https://blast.ncbi.nlm.nih.gov/Blast.cgi) of the GenBank nonredundant nucleotide or whole-genome sequence (WGS) databases with the left and right boundaries of AbaR4 (Table 2) in the MRSN56 chromosome (GenBank accession number CP080452) as the query (last searched 7^th^ May 2024). Further searches of the genomes were performed using the boundaries of AbaR28, Tn*7*, and Tn*7*+ (Table 2). Cutoffs of 99% identity and 100% coverage were applied. For each genome identified, the PubMLST database (www.pubmlst.org) was used to determine STs using the Pasteur MLST scheme and to identify *ampC* allele using the *ampC* database. Kaptive^36^ was used to identify KL and OCL loci for surface polysaccharide types. The location(s) of Tn*2006* (ISAba1-*oxa23*-ISAba1) in draft genomes was determined using a BLASTn with the ISAba1 sequence to retrieve pairs of contigs for chromosomal regions expected to be contiguous, that had inversely oriented remnants of ISAba1 at their end and characteristic 9 bp TSDs.

**Table 2:** Features and coordinates used to identify MRSN56 relatives

Feature	Coordinates in MRSN56^a^
Left-hand boundary of AbaR4	946987-947886
Right-hand boundary of AbaR4	963681-964580
Left-hand boundary of AbaR28 with chromosomal deletion	248495-249394
Tn*7*+ in *glmS -* unique boundary	3911750-3912649
Tn*7* in secondary location	2109334-2110233

^a^GenBank accession number CP080452.

### Core SNP phylogeny

A recombination-free approach was used to compare *A. baumannii* isolates in this study to determine their evolutionary relationship. Briefly, Illumina reads for all isolates were mapped to the MRSN56 reference using Snippy (https://github.com/tseemann/snippy) to generate a whole-genome alignment. High-quality variant sites were called using SAMtools v1.3.1.24^37^ with standard-quality filtering. Single nucleotide differences in recombinant regions were identified and removed using Gubbins v2.1.025^38^ with default parameters, including the default taxa filtering percentage of 25%. A maximum likelihood phylogenetic tree was inferred from the resulting recombination-filtered alignment using RAxML (v.8) with the GAMMA model.

### ISAba1 and ISAba125 mapping

ISMapper^25^ was used to map the locations of copies of ISAba1 in strains examined in this study. The complete genome of MRSN56 (GenBank accession no. CP080452) was used as the reference sequence. As this analysis uses reads as input, only draft genomes with reads available in the Sequence Read Archive (SRA) were examined. The sequences of ISAba1 and ISAba125, retrieved from ISFinder (https://isfinder.biotoul.fr/), were used as the query sequence, and paired-end Illumina reads were the input for the remaining isolates of interest. After mapping, the individual output text files were compiled using a compiled_table.py script and were sorted by prevalence. The individual IS locations in the chromosome identified by their location in the MRSN56 reference genome were compiled.

## Data Availability

The complete genomes of MRSN571146 and MRSN576822 have been deposited in GenBank (BioProject PRJNA761133) under BioSample accession numbers SAMN21245944 and SAMN21245945, respectively. A list of accession numbers of publicly-available genomes used in this study is provided in Table 2.
